# Spinal ependymoma: a comprehensive review of molecular classification, management guidelines, and clinical outcomes with a focus on the pediatric population (Part III of ependymomas across compartments)

**DOI:** 10.1007/s11060-026-05816-9

**Published:** 2026-10-06

**Authors:** Anthony M. Price, Nathan S. Fredricks, George Koutsouras, Frederic A. Vallejo, Christopher Bonfield, Devang J. Pastakia, Leo Y. Luo, Frederick Boop, Vijay Ramaswamy, Michael C. Dewan

**Affiliations:** 1 Department of Neurological Surgery, Vanderbilt University Medical Center, Nashville, TN USA; 2 Division of Pediatric Neurosurgery, Monroe Carell Jr Children’s Hospital at Vanderbilt University Medical Center, Nashville, TN USA; 3 John Sealy School of Medicine, The University of Texas Medical Branch at Galveston, Galveston, TX USA; 4 Department of Neurosurgery, The University of Texas Medical Branch at Galveston, Galveston, TX USA; 5 Department of Pediatrics, Vanderbilt University Medical Center, Nashville, TN USA; 6 Division of Pediatric Hematology/Oncology, Monroe Carell Jr Children’s Hospital at Vanderbilt University Medical Center, Nashville, TN USA; 7 Department of Radiation Oncology, Vanderbilt University Medical Center, Nashville, TN USA; 8 Department of Neurosurgery, University of Tennessee Health Science Center, Memphis, TN USA; 9 St. Jude Children’s Research Hospital, Memphis, TN USA; 10 Division of Haematology/Oncology, The Hospital for Sick Children, Toronto, ON Canada

**Keywords:** Ependymoma, Spinal ependymoma, Intramedullary tumor, Myxopapillary ependymoma, MYCN amplification, Laminoplasty, Pediatric spinal ependymoma

## Abstract

**Purpose:**

Spinal ependymomas are rare tumors of the central nervous system characterized by distinct anatomical, histological, and molecular subtypes. This review aims to synthesize current evidence regarding the presentation, diagnosis, surgical management, adjuvant therapy, and outcomes of spinal ependymomas, with a focus on the pediatric population.

**Methods:**

We conducted a narrative review of contemporary literature, consensus recommendations, and relevant treatment-related molecular classifications. Key topics included preoperative evaluation, neuraxis imaging, intraoperative strategies, extent of resection, histopathologic and molecular risk stratification, adjuvant radiotherapy and chemotherapy, recurrence patterns, and long-term surveillance. Representative clinical cases illustrating the surgical management of intramedullary spinal ependymomas and myxopapillary ependymomas are provided in the supplementary material.

**Results:**

Spinal ependymomas encompass spinal ependymoma, spinal ependymoma with MYCN amplification, spinal subependymoma, and spinal myxopapillary ependymoma. The primary therapeutic goal is maximal safe gross total resection, which is the most consistent modifiable predictor of progression-free survival. Molecular classification enhances risk stratification, especially for the aggressive MYCN-amplified subgroup. However, histologic grade and the extent of resection remain critical to management. Adjuvant radiotherapy is generally recommended for cases involving subtotal resection, grade 3 disease, tumor dissemination, capsular violation in myxopapillary tumors, or high-risk biology. Chemotherapy does not have a well-established role as a first-line treatment. Much of this evidence is extrapolated from adult or mixed-age cohorts, however, and pediatric-specific data remain limited across nearly every management domain, a gap we characterize systematically.

**Conclusion:**

Management of spinal ependymomas is centered around surgery and adapted to individual risk factors. Overall survival is generally favorable for spinal ependymomas, but recurrence risk remains substantial and varies by extent of resection and molecular subtype. Lifelong surveillance is crucial, as both local recurrence and craniospinal dissemination may occur long after treatment.

**Supplementary Information:**

The online version contains supplementary material available at https://doi.org/10.1007/s11060-026-05816-9.

## Introduction

**Fig. 1:**
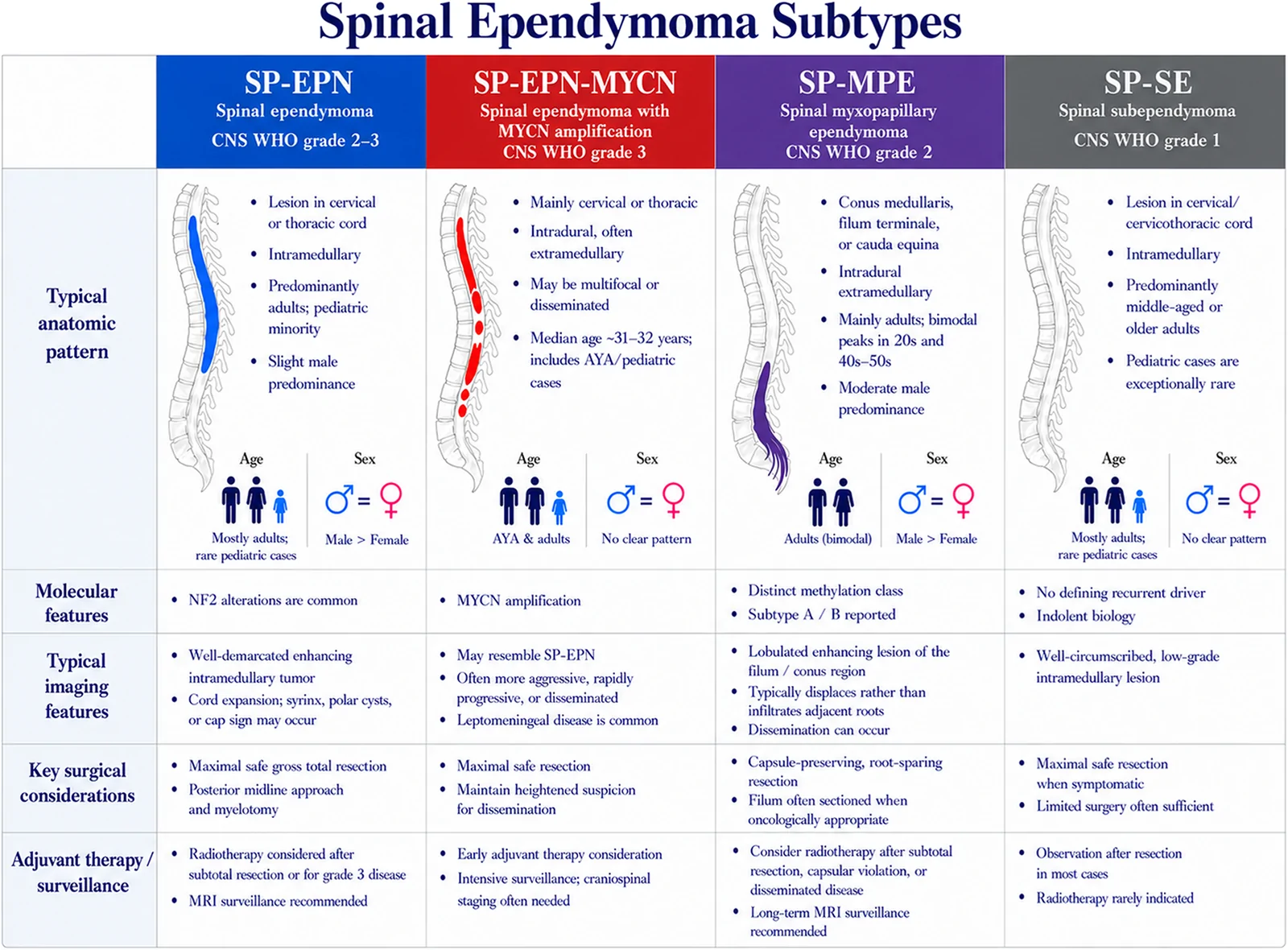
Spinal Ependymoma Subtypes. Summary of the major spinal ependymoma subtypes recognized in the WHO CNS5 classification, including spinal ependymoma (SP-EPN), spinal ependymoma with MYCN amplification (SP-EPN-MYCN), spinal myxopapillary ependymoma (SP-MPE), and spinal subependymoma (SP-SE); Abbreviations: CNS, central nervous system; WHO, World Health Organization; CNS WHO, World Health Organization Classification of Tumors of the Central Nervous System; SP-SE, spinal subependymoma; SP-EPN, spinal ependymoma; SP-EPN-MYCN, spinal ependymoma with MYCN amplification; SP-MPE, spinal myxopapillary ependymoma; MYCN, MYCN proto-oncogene; RT, radiotherapy

While intracranial ependymomas are more common in children, spinal ependymomas are predominantly found in adults [1–5]. Spinal ependymomas represent the most common intramedullary spinal cord neoplasm in adults, accounting for 50–60% of lesions; meanwhile, pediatric spinal ependymomas account for just 1% of all childhood CNS neoplasms [1–5]. Ependymomas found in the spinal compartment can be divided into four entities: spinal ependymoma (SP-EPN), spinal ependymoma with MYCN amplification (SP-EPN-MYCN), spinal subependymoma (SP-SE), and spinal myxopapillary ependymoma (SP-MPE) **(**Fig. 1**)** [6]. This clinical review outlines the unique treatment considerations and outcomes for all four subtypes with a particular focus on the pediatric population.

The primary goal of management is to achieve tissue diagnosis (including histopathological and molecular characterization), gross total resection (GTR), or the maximal safe surgical resection margin, which has been consistently linked to improved progression-free and overall survival rates [1, 3, 7]. For higher-grade spinal ependymomas such as SP-EPN-MYCN that are not completely resected, adjuvant radiotherapy (RT) is recommended [1, 3, 8]. As in intracranial ependymoma, the role of chemotherapy is limited and generally reserved for young children or cases of recurrent or metastatic disease [9–11]. Regardless of the treatment, patients require lifelong surveillance due to the risk of late recurrence and distant disease [1, 7]. This review compiles current literature and consensus recommendations to guide practical, evidence-based management of these lesions. A summary of the pediatric-specific vs. adult-extrapolated evidence for these spinal ependymomas is compiled in Table 1. A non-systematic selection of landmark studies is summarized in Supplementary Table 1.

**Table 1:** Clinical decision-making for spinal ependymoma, with recommendation, supporting evidence, and evidence type by domain — highlighting where practice is pediatric-specific, extrapolated from adult/mixed-age data, or unaddressed

Clinical Decision	Recommendation	Evidence Basis	Evidence Type
**Clinical Presentation and Imaging**			
Diagnostic presentation and delay	Maintain a low threshold for neuraxis MRI with nonspecific signs in preverbal children (torticollis, motor regression, refusal to walk/crawl) — these precede diagnosis by longer intervals than adult presentations.	Pediatric case series document this delayed, nonspecific presentation pattern [23]	Pediatric-direct
Surveillance imaging under sedation/anesthesia	Balance lifelong surveillance intervals against cumulative anesthesia exposure; no disease-specific protocol yet defines the optimal interval.	General pediatric anesthesia/neuroimaging literature raises neurodevelopmental concerns with repeated exposure [24]; no ependymoma-specific interval data exist.	Extrapolated (risk) + No data (interval)
**Surgical Management**			
GTR as primary treatment goal	Maximal safe GTR remains the primary goal in children, as in adults.	Pediatric/AYA series confirm GTR predicts PFS [8, 28, 66, 67], directionally consistent with the larger adult-dominant literature [4, 43, 49, 50]. A systematic review comparing adult and pediatric SP-MPE outcomes directly found distinct age-based patterns, showing surgical outcome data cannot be assumed to transfer uniformly [52].	Pediatric-direct; Outcomes diverge by age for SP-MPE
Laminoplasty vs. laminectomy for kyphosis prevention	Don’t choose laminoplasty on the assumption it prevents deformity — it doesn’t. Base fusion decisions on risk factors, not on approach.	Pediatric cohorts directly comparing the two techniques found no protective benefit of laminoplasty [34–38]; a mixed-age review reaches the same conclusion [39]. Some surgeons favor laminoplasty for ≥ 3-level exposures for unproven CSF-leak- and reoperation-landmark-related reasons; the only comparative CSF-leak data available found no significant difference between techniques [42].	Pediatric-direct (deformity outcome); No data (CSF-leak/reoperation rationale — practice pattern only, one small series against)
Posterior tension-band–preserving technique	May offer a deformity-mitigation option, but counsel families it is unvalidated.	Mechanism is plausible; evidence is limited to a single case report [40].	No data (case-report level only)
Instrumented fusion — indication and timing	Reserve prophylactic fusion for children with multiple risk factors rather than routine use; a low threshold is reasonable in high-risk children. When fusion is needed, prefer MRI-compatible implants or constructs planned for removal once fusion is solid.	Pediatric series define risk factors and complication rates [36, 37, 41]; no trial compares prophylactic vs. reactive strategies. Permanent hardware can compromise surveillance MRI [38]; the preference for MRI-compatible or removable constructs reflects expert practice, not comparative data.	Pediatric-direct (risk factors) + No data (strategy) + Expert opinion (hardware choice)
Intraoperative neurophysiological monitoring	Continue treating multimodal IONM as standard despite a minority challenge to the paradigm.	The challenge comes from an adult-dominant series [50]; a separate multimodal-IONM cohort reports favorable long-term neurologic outcomes without resolving the debate [57]. Pediatric-specific monitoring accuracy has never been separately established.	Extrapolated (challenge) + No data (pediatric performance)
**Radiation Therapy**			
Timing/deferral of RT in young children	Treat deferral as an unproven strategy borrowed from intracranial protocols — use only within a multidisciplinary/trial context.	No spinal-ependymoma study has tested RT timing/deferral; rationale is borrowed entirely from intracranial trials [81].	No data (spinal-specific)
Dose selection, grade 2 SP-EPN/localized disease	Use focal conformal RT (45–59.4 Gy) for STR/biopsy, following adult/mixed-age dosing thresholds.	Dosing is extrapolated from adult/mixed-age series, including the original prospective conformal-RT trial establishing local-control rates [49, 66, 72]; a pediatric-focused review synthesizes indications but not independent dosing [71].	Extrapolated (dosing) + Pediatric-direct (indications only)
Outcomes, high-grade/non-myxopapillary SP-EPN	Proton RT at 50–54 Gy is reasonable, favored for normal-tissue sparing.	A bicentric pediatric proton-therapy series directly supports this dose range in children [76].	Pediatric-direct (single bicentric series)
Outcomes, high-risk SP-MPE	Maintain surveillance beyond the RT field and intracranially even after excellent in-field control.	A pediatric/YA-focused multi-institutional series directly demonstrates this pattern [75]; adult-dominant series describe RT’s disease-control role more generally [73, 74], and dissemination/metastasis management is addressed separately [16].	Pediatric-direct
Radiation-induced spinal growth deformity	Counsel that therapeutic RT doses substantially exceed deformity thresholds in young children; weigh explicitly against surgical deformity risk, especially under age 6.	A comprehensive PENTEC review defines dose-dependent thresholds by age [77]; biology is disease-agnostic but the cohort isn’t ependymoma-specific (footnote a).	Pediatric-direct (biology), not disease-specific
**Chemotherapy**			
Frontline chemotherapy role	Do not use chemotherapy as frontline therapy outside recurrence, dissemination, or trial enrollment.	The pivotal intracranial trial shaping this recommendation excluded primary spinal cord tumors [79, 80]; a related protocol addressing chemotherapy for incompletely resected disease is similarly intracranial-dominant [78].	Extrapolated
Infant RT-deferral (chemo-as-bridge) strategy	Reasonable to extend to spinal disease, but frame to families as unproven in this compartment.	Validated only in pediatric intracranial ependymoma [81]; never formally studied in spinal disease.	Extrapolated
**Molecular Classification and Outcomes**			
Risk stratification, SP-EPN-MYCN	Perform reflex MYCN testing on all spinal specimens; a positive result triggers high-risk counseling, increased surveillance, and trial-enrollment consideration.	Pediatric/AYA-inclusive cohorts directly establish this subgroup’s aggressive spinal behavior [13, 15, 18]; a separate methylation-confirmation study supports the distinct molecular signature [68]. Intracranial risk-stratification models offer extrapolated context [82].	Pediatric-direct
Long-term survival/overall outcomes	Counsel that survival is generally favorable but recurrence risk is durable — lifelong surveillance is warranted regardless of initial response.	Multiple pediatric-inclusive cohorts directly document this pattern [20, 66, 71, 84, 85].	Pediatric-direct
**Neurofibromatosis Type 2**			
NF2 surveillance and management	Apply standard NF2 tumor-surveillance protocols; no ependymoma-specific modification is currently supported.	NF2-ependymoma association and surveillance are established across mixed pediatric/adult NF2 cohorts [85–89].	Pediatric-inclusive, not spinal-ependymoma-specific

**Abbreviations**: AYA, adolescent/young adult; GTR, gross total resection; IONM, intraoperative neurophysiological monitoring; NF2, neurofibromatosis type 2; PENTEC, Pediatric Normal Tissue Effects in the Clinic; PFS, progression-free survival; RT, radiotherapy; SP-EPN, spinal ependymoma; SP-EPN-MYCN, spinal ependymoma with MYCN amplification; SP-MPE, spinal myxopapillary ependymoma; STR, subtotal resection; YA, young adult. Evidence Type reflects whether the cited data derive directly from pediatric/AYA cohorts (“Pediatric-direct”), are borrowed from adult or mixed-age literature (“Extrapolated”), or do not yet exist for this population (“No data”)

^a^ Dose-response data for radiation-induced spinal growth deformity are pooled from mixed pediatric radiation-oncology populations (predominantly other pediatric solid tumors requiring spinal irradiation), not from an ependymoma-specific cohort; the underlying vertebral growth-plate biology is disease-agnostic and the reported thresholds are expected to transfer directly

## Molecular landscape

The most recent fifth edition of the 2021 World Health Organization (WHO) Classification of Tumors of the Central Nervous System (CNS) guidelines defines two spinal ependymoma subtypes and two further subtypes that are not exclusive to but commonly occur in the spinal canal: spinal ependymoma (SP-EPN), spinal ependymoma with MYCN amplification (SP-EPN-MYCN), spinal subependymoma (SP-SE), and spinal myxopapillary ependymoma (SP-MPE). Integrated diagnosis is no longer based on histology alone but now relies on combined anatomic compartment, histomorphology, and molecular features. For example, the 2021 upgrade of SP-MPE from grade 1 to grade 2 recognizes that incompletely resected tumors historically managed with observation alone can recur with subarachnoid dissemination, making these difficult to treat and typically requiring craniospinal irradiation, carrying substantial morbidity [6, 12–16]. 

Pediatric-specific data add important insights. For example, in the HIT-MED spinal cohort, which included 83 patients aged 22 years or younger, the distribution of methylation classes was as follows: SP-MPE (63%), SP-EPN (33%), and MYCN-amplified (4%) [13]. Within this cohort, the extent of surgical resection and WHO grade had a significant impact on progression-free survival. However, molecular class (comparing SP-MPE and SP-EPN) did not clearly differentiate risk levels in this pediatric population. Therefore, while molecular profiling refines biological classification, surgical resection and histologic grade remain the primary determinants of outcomes for most children.

The molecular classification behind SP-EPN-MYCN class represents one of the most aggressive subgroups and may be the exception [15, 17]. Tumors in this category often show early relapse (typically within 2.3 years) and can lead to death despite aggressive treatment despite age at diagnosis. Consequently, identifying MYCN amplification has immediate clinical implications, such as high-risk counseling, increased craniospinal surveillance, and consideration of participation in clinical trials or novel therapeutic approaches.

Within pediatric SP-MPE, methylation subtype B has shown a trend toward improved 5-year progression-free survival when compared to subtype A (86% vs. 56%), with subtype A being more frequently disseminated. However, these differences have not reached statistical significance, and strategies for de-intensification of MPE-B remain under investigation [13]. Importantly, even lesions typically perceived as indolent, such as MPE, carry a substantial long-term risk of recurrence, with a 10-year progression-free survival rate of approximately 59% in mixed-age datasets [17, 18]. 

SP-SE is a rare, indolent CNS WHO grade 1 tumor more common in adults than children. Symptomatic or progressive lesions are managed with maximal safe resection, while observation is reasonable for stable asymptomatic disease; adjuvant radiotherapy is rarely indicated [9, 19]. 

## Clinical presentation

The neurosurgical management begins with a firm understanding of the nature and time course of symptom onset and a detailed neurological exam, paying special attention to motor and/or sensory long-tract disruptions and urodynamic changes. Spinal ependymomas often present in patients with symptoms of progressive sensory disturbances, gait imbalance, motor weakness, axial back pain, or radicular pain [2, 7, 20]. Despite this array of symptoms, most patients are still ambulatory at diagnosis. Symptoms typically develop gradually, which can lead to delayed diagnoses and extensive spinal cord involvement by the time spinal ependymomas are identified [21, 22]. Rapid deterioration is rare, barring hemorrhage of the mass.

In young children, presentation is often nonspecific, and diagnosis is correspondingly delayed relative to adults, who can more reliably localize and describe symptoms. Torticollis, regression of previously acquired motor milestones, refusal to walk or crawl, and irritability may be the only signs in a preverbal child, and are easily misattributed to more common musculoskeletal or developmental causes [23]. 

## Neurological imaging

If not already obtained, the clinical findings above necessitate total neuraxis magnetic resonance imaging (MRI) with gadolinium. Even when imaging suggests an isolated spinal lesion, it is crucial to also obtain a contrast-enhanced MRI of the brain to rule out synchronous intracranial or leptomeningeal disease. Spinal ependymomas as drop metastases occur in a minority of patients; however, their presence is clinically significant and will significantly impact staging and the planning of adjuvant therapy. Therefore, failing to perform complete neuraxis imaging may lead to under-staging of disease burden and may hinder adequate postoperative management [2, 9, 19]. High-resolution T2-weighted steady-state sequences help identify the tumor-cord interface and guide the optimal site for myelotomy.

Obtaining adequate imaging carries its own pediatric-specific burden: young children frequently require sedation or general anesthesia to complete a diagnostic-quality MRI, and because lifelong surveillance imaging is recommended for this disease, children face repeated anesthetic exposure at an age when its neurodevelopmental safety remains incompletely characterized [24]. This consideration does not change the imaging indication but should inform discussions with families and anesthesia teams about surveillance intervals.

At this stage, differential diagnoses remain compartment-based. Intramedullary SP-EPN, SP-EPN-MYCN, and SP-SE should be distinguished from astrocytomas, hemangioblastomas, cavernous malformations, and inflammatory or demyelinating lesions. Extramedullary conus-filum SP-MPE should be distinguished from schwannomas, meningiomas, paragangliomas, drop metastases, or arachnoiditis.

### MRI characteristics of spinal ependymomas (SP-EPN), spinal ependymomas with MYCN amplification (SP-EPN-MYCN), and spinal subependymoma (SP-SE)

**Fig. 2:**
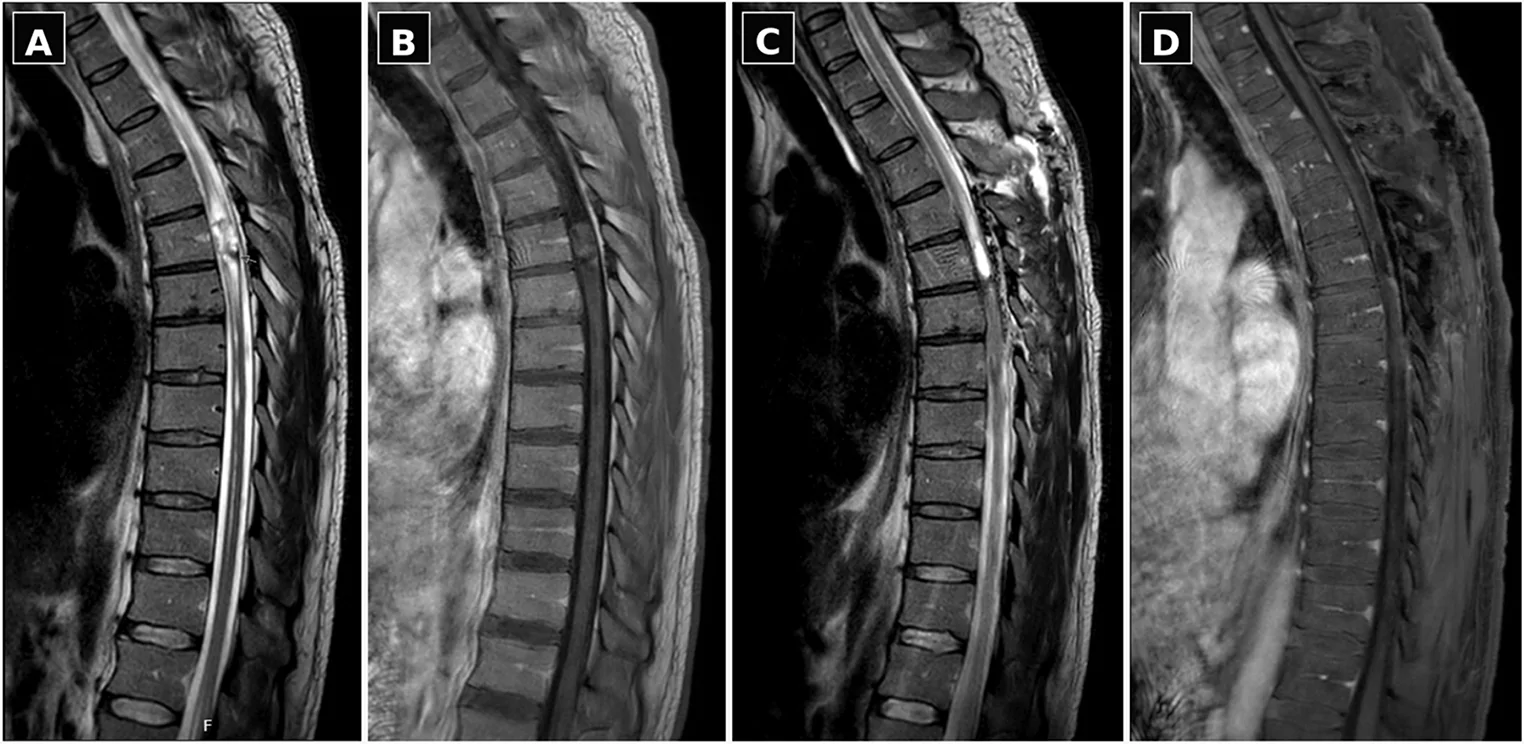
Preoperative and postoperative sagittal MRI of a Thoracic Spinal Ependymoma (SP-EPN): (**A**) Preoperative sagittal T2-weighted MRI demonstrates a focal intramedullary ependymoma in the thoracic spinal cord with associated cord expansion, (**B**) Preoperative sagittal T1-weighted post-contrast MRI demonstrates associated enhancement at the level of the lesion, (**C**) Postoperative sagittal T2-weighted MRI demonstrates expected postoperative changes following tumor resection with decompression of the thoracic spinal cord, and a T4–T6 laminoplasty, (**D**) Postoperative sagittal T1-weighted post-contrast MRI demonstrates no residual enhancing lesion, consistent with gross total resection, with reconstruction of the T4–T6 posterior elements through laminoplasty

SP-EPN and SP-EPN-MYCN typically are intramedullary and have a well-defined tumor-cord interface, suggesting a higher likelihood of achieving GTR **(**Fig. 2**)** [7, 20, 25]. SP-EPN, SP-EPN-MYCN, and SP-SE typically occur in cervical or cervicothoracic locations [26]. These three subtypes often demonstrate symmetric cord expansion, well-demarcated margins, and contrast enhancement [21, 22]. Additionally, polar cysts are common features, and 40–70% of cases present with syringomyelia. The presence of a syrinx is not inherently a negative prognostic indicator; rather, it may help facilitate the identification of a surgical dissection plane during surgery [7, 27]. Finally, a peripheral rim of hemosiderin deposition, known as the “cap sign,” may be present at one or both poles. Collectively, these features support the presence of a distinct gliotic plane that can be utilized for surgical dissection [25, 28]. While MYCN amplification cannot be determined through radiographic imaging, unusually aggressive imaging features, rapid progression, or evidence of dissemination should raise clinical suspicion for high-risk molecular disease [12–15]. SP-EPN-MYCN may display aggressive imaging features like irregular or poorly defined margins, infiltrative extensions, multifocal nodularity, or leptomeningeal enhancement.

### MRI characteristics of spinal myxopapillary ependymomas (SP-MPE)

**Fig. 3:**
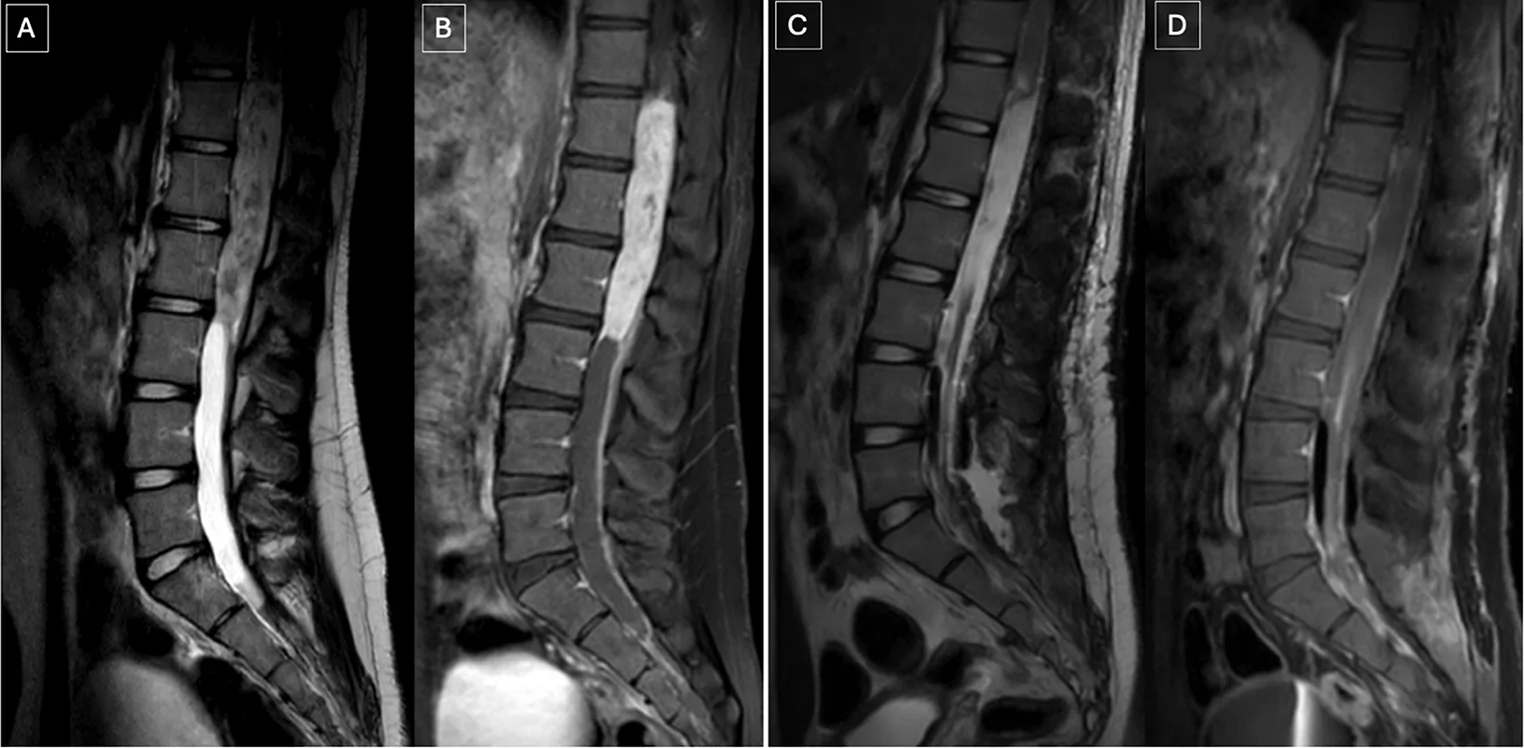
Preoperative and postoperative sagittal MRI of a Myxopapillary Ependymoma (SP-MPE): (**A**) Preoperative sagittal T2-weighted MRI demonstrates a heterogeneous expansile lesion involving the lower spinal cord/conus region, (**B**) Preoperative sagittal T1-weighted post-contrast MRI demonstrates associated intralesional enhancement, (**C**) Postoperative sagittal T2-weighted MRI demonstrates expected postoperative changes following tumor resection and decompression of the distal thecal sac, (**D**) Postoperative sagittal T1-weighted post-contrast MRI demonstrates no residual enhancing lesion, consistent with gross total resection, with postoperative laminoplasty changes

Contrary to the types above, SP-MPE exhibit distinct radiographic characteristics, typically arising in an extramedullary location or at the filum terminale, often with a lobulated morphology and significant contrast enhancement **(**Fig. 3**)** [25]. These lesions are more likely to displace rather than infiltrate adjacent neural elements, although they carry a risk of cerebrospinal dissemination.

## Surgical management

**Fig. 4:**
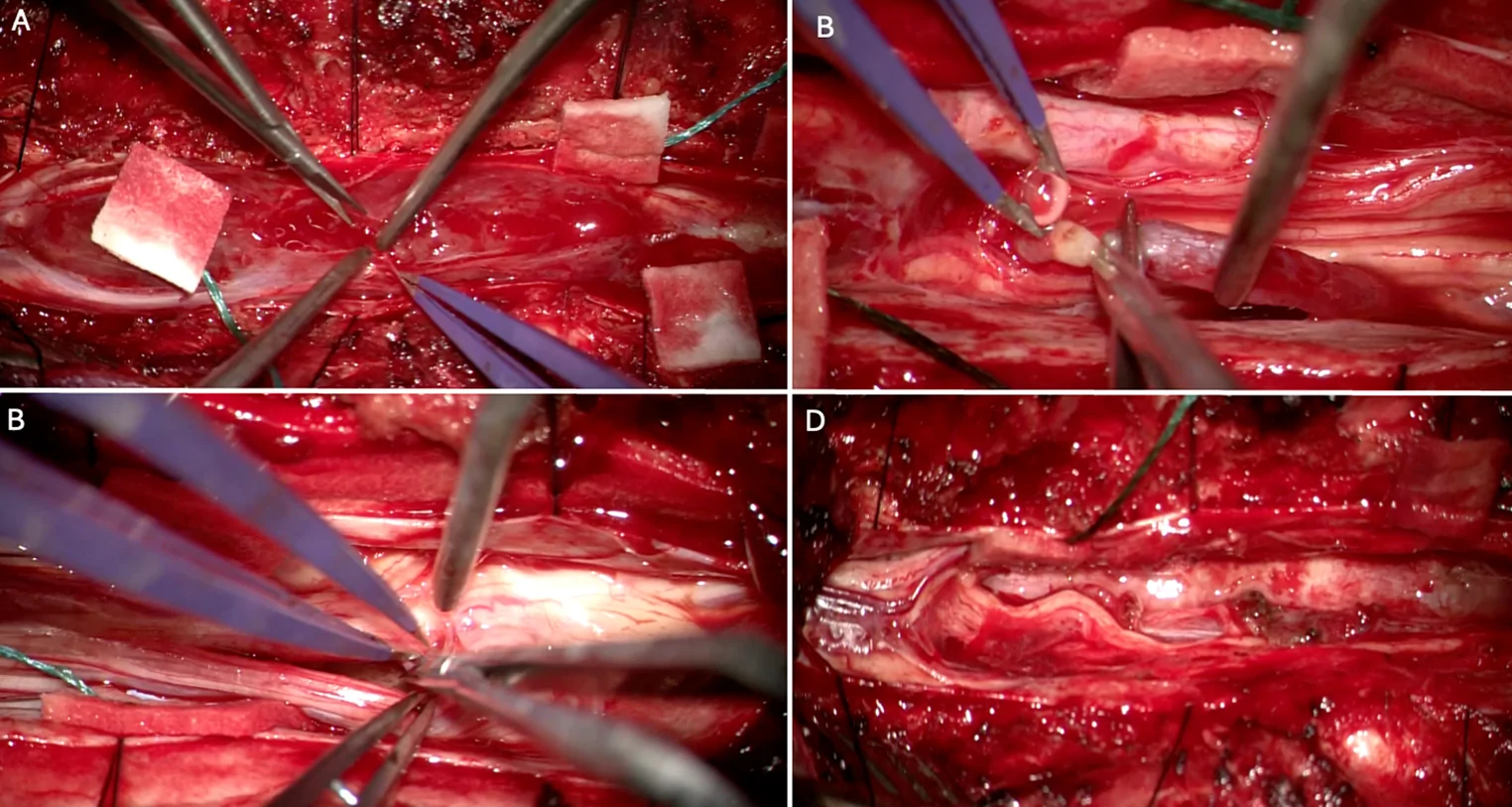
Intraoperative appearance and resection of a lower spinal cord/filum terminale–associated myxopapillary ependymoma (SP-MPE): (**A**) Initial exposure demonstrates a hemorrhagic and friable-appearing SP-MPE, (**B**) The filum terminale is identified and sectioned, serving as a key anatomic landmark for maintaining orientation to the conus medullaris and distal spinal cord throughout tumor resection, (**C**) The white, glistening cystic tumor capsule is visualized with dense adherence to the cauda equina nerve roots; the capsule is dissected free where safely separable, although aggressive removal of densely adherent cyst wall may not be necessary when preservation of neural elements is prioritized, (D) Final operative view after resection demonstrates the conus medullaris, displaced cauda equina nerve roots, and ventral dura

The primary goals of surgery include tissue diagnosis (including histopathological and molecular characterization) and maximal safe resection. The surgeon must integrate these goals with their preoperative assessment along three complementary fronts: (1) the feasibility of GTR, (2) the functional neurologic risks of GTR, and (3) the biological risk for tumor recurrence or dissemination. Factors influencing anatomic feasibility of GTR include the extent of the lesion, the rostrocaudal levels, tumor-cord interface, associated cysts and syringomyelia, and any signs of dissemination present on radiologic studies [10, 12, 13, 17, 27]. The neurological risk is influenced by the patient’s baseline neurological exam, the current degree of spinal cord expansion, the tumor’s proximity to spinal tracts, and the patient’s ability to tolerate aggressive surgical maneuvers, as judged by intraoperative neuromonitoring (IONM) [21, 22, 29–31]. Finally, the biological risks are influenced by the subtype of ependymoma, the histologic grade, any radiographic evidence of dissemination, and potential capsular violations, particularly in cases of SP-MPE [12–15, 17] (Fig. 4). 

### Surgical approach and exposure

The operative setup and approach for spinal ependymoma are guided by the three fronts noted above. Most SP-EPN and SP-EPN-MYCN lesions are central and intramedullary, leading to symmetric cord expansion that often spans multiple vertebral segments. A posterior midline approach is the standard for these lesions [22, 32]. Therefore, surgical exposure must allow for adequate visualization to enable safe microsurgical dissection while preserving long-term spinal stability, particularly in young patients with continued skeletal growth. Whether a laminectomy or laminoplasty is performed, the operator must take care not to disrupt the facet joints laterally [21, 22, 33]. In cases requiring extensive multilevel resection, where there is disruption of the facet joints, compromise of the posterior ligamentous complex, or pre-existing deformity, instrumented fusion may be required to maintain structural alignment, although there is conflicting evidence on whether laminoplasty vs. laminectomy is better for kyphosis prevention [34]. 

Outcomes across both techniques are consistently poor rather than genuinely conflicting, and postoperative spinal deformity is arguably the most important surgical morbidity in the pediatric population specifically [35]. McGirt et al. found no difference in deformity rates between laminoplasty and laminectomy overall (9% vs. 12%), and in patients under 18 years the rates rose to 43% and 36% respectively — several times the overall rate, with no protective benefit from laminoplasty [35]. What is well established is who is at risk. In 161 children, Yao et al. found that 27% eventually required fusion, with risk rising with age under 13 (OR 4.4), preoperative scoliosis (OR 3.2), a tumor-associated syrinx (OR 3.4), thoracolumbar junction involvement (OR 2.6), and each additional resection (OR 1.8); children who required fusion had worse long-term McCormick and Karnofsky scores [36]. Ahmed et al. independently identified exposure beyond four levels as a predictor of fusion need [37]. Most strikingly, Hogg et al., in 45 children operated with laminoplasty throughout, found deformity present in 38% already at presentation and in 69% postoperatively, with 31% eventually undergoing fusion — underscoring that deformity risk in this population is driven substantially by tumor biology and preoperative status, not surgical technique alone [38]. These findings argue for a low threshold for prophylactic instrumented fusion in children with multiple risk factors, particularly age under 13, more than four levels of exposure, preoperative scoliosis, thoracolumbar junction involvement, or a tumor-associated syrinx.

The rationale for choosing laminectomy, laminoplasty, or adding instrumented fusion in a child reflects a genuine trade-off rather than a settled preference. Laminoplasty is intended to preserve the posterior elements and reconstruct the laminar arch over the dura, and is often favored in children for this theoretical benefit; however, its actual impact on long-term spinal alignment compared with laminectomy remains uncertain, and as above, neither technique reliably prevents deformity [39]. The posterior ligamentous complex disrupted during either approach has a tendency not to heal, which is thought to contribute to progressive kyphosis; techniques such as in situ laminotomy, which preserve this tension band while still exposing the canal, have been proposed specifically to address this mechanism, though evidence is currently limited to case reports [40]. Instrumented fusion reliably restores stability once extensive bone removal (e.g., corpectomy, multilevel facetectomy) or established deformity make the spine unstable, but in a growing child it permanently eliminates motion and growth potential at the fused levels. A systematic review found concomitant fusion at the time of tumor resection is infrequently performed in children, is most often reserved for extensive bone removal or severe baseline deformity rather than used prophylactically, and carries its own risks — CSF leak and new neurological deficit were each reported in roughly one in ten cases — with insufficient evidence to determine whether routine use is safe or beneficial [41]. Instrumentation carries a further cost in this disease: children with ependymoma need lifelong surveillance MRI, and hardware artifact can compromise tumor surveillance [38]. Where fusion is unavoidable, MRI-compatible implants that allow visualization of the spinal cord, or constructs planned for removal once fusion is solid, are preferable, though this reflects expert practice rather than comparative data. In practice, this leaves a genuine judgment call between prophylactic fusion in a high-risk child to avoid a second surgery, versus reserving fusion for those who progress to symptomatic deformity under close surveillance.

In the authors’ practice, laminoplasty is generally favored over laminectomy when exposure of three or more levels is required, for two theoretical reasons that have not been substantiated in the literature and warrant dedicated study. First, restoring the lamina may mitigate postoperative CSF leak by supporting the multilayer closure and reducing dead space in the dorsal epidural space — though the only comparative data available, a 94-patient mixed-age series, found no significant difference in CSF leak rate between laminoplasty and laminectomy [42]. Second, the presence of lamina at reoperation (e.g., for recurrent disease) may provide a useful anatomic landmark and reduce the risk of inadvertent dural injury during re-exposure; this is consistent with the well-established finding that prior laminectomy is a risk factor for incidental durotomy at revision spine surgery generally, though no study has tested this specifically in the tumor-recurrence setting.

### Extent of resection

GTR is the most consistent and modifiable predictor of progression-free survival in spinal ependymoma [2, 3, 7, 28, 43, 44]. For SP-EPN and SP-EPN-MYCN lesions, a posterior median sulcus myelotomy provides the most anatomically appropriate corridor, with the dissection plane often defined by a gliotic margin from prior hemorrhage or syringomyelia. Early internal debulking or cyst decompression facilitates safer, lower-traction dissection, with small anterior perforating vessels isolated and divided carefully to avoid vascular compromise [45–48]. 

Reported GTR rates in contemporary series range from approximately 72–83% [4, 43, 49]. However, these numbers are not uncontested: Sweeney et al. reported a 94% GTR rate using en-bloc resection without intraoperative neurophysiological monitoring (IONM), arguing that series reporting lower resection rates may reflect conservative responses to false-positive monitoring alarms rather than a true ceiling on safe resection [50]. This remains a minority position: most contemporary series continue to treat multimodal IONM as standard given the potentially irreversible cost of a false negative, but it represents a genuine point of disagreement in the surgical literature that merits acknowledgment rather than omission.

Specific surgical considerations differ for SP-MPE [45, 51–53]. These tumors are usually centered on the filum terminale or cauda equina and are extramedullary. The filum is often intrinsically incorporated into the mass and, when appropriate, should be sectioned and removed with the specimen to facilitate complete resection. Because the operative task is typically one of meticulous separation of the tumor capsule from the surrounding lumbosacral roots rather than from spinal cord parenchyma, electromyographic nerve root monitoring is indicated. Capsule preservation is especially important in SP-MPE. Although the literature is heterogeneous, several series associate capsule violation with increased local recurrence, cerebrospinal fluid dissemination, and a greater likelihood of adjuvant radiotherapy [54, 55]. Accordingly, en bloc resection is preferred when technically feasible, especially for filum-based lesions [54, 55].

### Intraoperative surgical adjuncts and postoperative considerations

Intraoperative adjuncts play a crucial role in maximizing the extent of resection while minimizing risks of permanent neurological complications [29–31, 56–60]. Continuous multimodal IONM, including somatosensory evoked potentials (SSEPs), transcranial motor evoked potentials (MEPs), and D-wave monitoring when available, allows for real-time assessment of the integrity of the dorsal column and corticospinal tract. While isolated changes in SSEPs during posterior myelotomy should be interpreted with caution, a sustained deterioration in MEPs or a significant reduction in D-wave amplitude is strongly associated with an increased risk of permanent motor deficits [61]. In cases of irreversible signal compromise, subtotal resection (STR) may be the appropriate approach. For MPE with clear separation from the distal conus, electromyography monitoring may be sufficient.

Intraoperative ultrasound is another helpful adjunct for confirming the localization of the lesion before dural opening and myelotomy [21, 31, 62, 63]. It helps define the cranial and caudal poles of the tumor, identify associated polar cysts or syrinxes, and assess for residual disease after internal debulking [64].

Early postoperative MRI (within 24–48 h) should assess the extent of resection and rule out potentially postsurgical complicating features [21, 32, 65]. In cases of STR, particularly with focal residual disease that is surgically accessible, early second-look surgery should be considered prior to adjuvant therapy [4, 49, 66, 67]. If available, intraoperative MRI performed prior to closure can save a return visit to the OR in patients with radiographic residual – and resectable – disease. Pathologic evaluation should include formal histologic grading in accordance with the WHO 2021 criteria, supplemented by DNA methylation profiling where available, and reflex testing for MYCN amplification in all spinal ependymoma specimens [13, 15, 26, 68].

## Radiation therapy

Adjuvant treatment decisions are still driven predominantly by histologic grade, extent of resection, and dissemination. Integrated molecular classification provides additional risk stratification; however, both the National Comprehensive Cancer Network (NCCN) and European Association of Neuro-Oncology (EANO) guidelines are currently based on grading [9, 13, 19, 69]. 

For localized CNS WHO grade 2 SP-EPN after GTR, most contemporary reports support surveillance without routine postoperative radiotherapy, as a clear benefit for adjuvant focal radiotherapy in GTR has not been consistently demonstrated [9, 19, 49, 66, 67]. By contrast, after STR or biopsy, focal conformal radiotherapy is recommended because incomplete resection is consistently associated with inferior progression-free survival, and improved local control has been reported with postoperative irradiation compared with surgery alone [9, 19, 49, 66, 70–72]. 

For grade 2 SP-MPE, surgery remains the key therapeutic intervention, but the role of adjuvant radiotherapy is more controversial [19, 54, 71]. Several retrospective series, including pediatric cohorts, suggest improved progression-free survival or local control with adjuvant radiotherapy even after gross total resection, particularly when dose escalation above 50.4 Gy is feasible or when capsule violation, multifocal disease, or dissemination are present [54, 55]. Other series, especially adult-dominant datasets with en bloc gross total resection, support observation alone after complete removal [55]. Given the rarity of SP-MPE, evidence for postoperative radiotherapy remains limited and heterogeneous: retrospective series suggest a disease-control benefit after subtotal resection, capsular violation, multifocal disease, dissemination, or other high-risk features — the same factors that most strongly favor adjuvant focal radiotherapy in practice — though population-level analyses have not shown a clear overall survival benefit [19, 54, 71, 73, 74]. 

A more recent multi-institutional series focusing specifically on pediatric and young adult high-risk MPE reported 5-year overall survival and progression-free survival of 100% and 60.8%, respectively, with low in-field local progression — but noted that patients remained at risk for recurrence outside the treated field and intracranially even when in-field control was excellent, arguing for continued vigilance beyond the treated volume rather than dose escalation alone [75]. 

For grade 3 SP-EPN, focal radiotherapy is generally recommended regardless of the extent of resection, given the higher risk of early recurrence [9, 19, 70]. SP-EPN-MYCN should be approached as a distinct, very high-risk subtype characterized by frequent grade 3 histology, leptomeningeal dissemination, early relapse, and poor survival despite repeated surgery, radiotherapy, and multi-agent chemotherapy [13, 15, 68]. Craniospinal irradiation should be considered when dissemination is present or strongly suspected [9, 13, 19]. Typical focal RT doses range from approximately 45 to 59.4 Gy, depending on tumor level and clinical context. For disseminated disease, CSI of approximately 36 Gy with a boost to gross disease to 45–59.4 Gy may be considered [9, 19, 70, 71]. 

Pediatric-specific outcome data for non-myxopapillary, high-grade spinal cord ependymoma are limited but growing: a bicentric proton therapy series in children found no tumor progression at a median follow-up of 6.3 years using total doses of 50–54 Gy, with radiation contributing only one additional neurologic deficit beyond those already caused by surgery — supporting this dose range’s safety in children and proton therapy’s dosimetric advantage in sparing normal tissue, including the growing vertebrae discussed above [76]. 

It is worth stating plainly that the grade- and subtype-stratified dosing and timing recommendations described above are derived almost entirely from adult and mixed-age literature. No prospective pediatric-specific data address whether these thresholds, or the timing of radiotherapy relative to ongoing skeletal and neurologic development, should differ in children, particularly the youngest patients in whom deferring radiotherapy could plausibly carry both benefit and risk. This gap is discussed further below.

Radiotherapy in children also carries a distinct, dose- and age-dependent risk not shared by adult patients: radiation to the growing spine can itself cause scoliosis and growth impairment, independent of any surgical contribution to deformity. A comprehensive PENTEC review of 603 pediatric patients found clinically significant scoliosis with vertebral doses as low as 15 Gy in infants under 2 years, with rates exceeding 30% above 20 Gy in children aged 2–6 years; children over 6 remained at risk above 30 Gy, generally with milder deformity [77]. The focal (45–59.4 Gy) and craniospinal (36 Gy plus boost) doses discussed above substantially exceed these thresholds, meaning radiation-induced spinal deformity should be weighed alongside surgical deformity risk when counseling families of young children — and is precisely why the timing question raised above is clinically urgent rather than theoretical.

It bears emphasis that this dose-response relationship derives from pooled pediatric radiation-oncology populations (predominantly other pediatric solid tumors requiring spinal irradiation) rather than an ependymoma-specific cohort. The underlying biology of the growing vertebra, however, is disease-agnostic, and the dose thresholds above apply regardless of the tumor being treated.

## Chemotherapy

For the spinal subtypes, chemotherapy has no established frontline role [9, 19, 78]. Systemic therapy is best considered in the setting of recurrent disease, dissemination, or clinical trials, where platinum- and temozolomide-based regimens and other experimental targeted approaches have been explored [9, 10, 19, 78]. However, because ACNS0831 excluded primary spinal cord tumors, these findings should be interpreted as supportive evidence for the limited role of chemotherapy in ependymoma rather than spinal-specific evidence [79, 80]. 

In very young children specifically, chemotherapy is sometimes used as a deliberate strategy to delay radiotherapy until the child is older and the developing CNS and spine are less vulnerable to radiation injury — an approach validated for pediatric intracranial ependymoma in trials such as SJYC07 [81], though it has not been formally studied in spinal disease and its extrapolation here is unproven.

## Recurrent disease

Local recurrence is the predominant pattern of failure in spinal ependymoma, even after GTR, while craniospinal dissemination occurs less frequently but is observed both at presentation and at relapse [9, 19, 71]. Functional outcomes are generally favorable with timely diagnosis and careful surgical technique; however, subtle motor, sensory, gait, and sphincter deficits remain common and require multidisciplinary rehabilitation support [2, 20, 67]. Given the risk of both early and very late recurrence, lifelong MRI surveillance is recommended for all patients, with increased frequency—including periodic brain and full neuraxis imaging—in those with dissemination or high-risk subtypes such as SP-MPE and SP-EPN-MYCN [9, 19, 20]. For isolated local recurrence, the dominant management strategy is repeat maximal safe resection when anatomically feasible, followed by focal radiotherapy in radiation-naive patients or carefully selected focal reirradiation after prior RT, with attention to cumulative spinal cord, conus, and cauda equina dose tolerance [9, 19, 71]. For disseminated or metastatic recurrence, craniospinal irradiation is the mainstay of salvage therapy in radiation-naive patients, with dose and field individualized to prior treatment and site of relapse; systemic therapy may be considered as an adjunct in refractory or heavily pretreated disease, though its benefit remains unproven [9, 19, 70, 71]. 

This dose-tolerance consideration is especially consequential in children, who may accumulate radiation exposure to the same at-risk levels across a much longer expected lifespan than an adult presenting with recurrence, and who carry the added risk of radiation-induced spinal growth deformity described above [77]. 

## Future directions

Several practical take-home points emerge from this review. Clinically, spinal ependymomas most often present with insidious signs of myelopathy, and complete neuraxis contrast MRI is required to characterize the extent of the pathology [2, 9, 19, 20]. Diagnostically, MRI features such as the tumor-cord interface, syrinx, and cap sign help distinguish the four spinal subtypes and potential resectability, though definitive classification requires histopathologic and molecular confirmation [7, 20, 25, 28, 63]. Therapeutically, maximal safe GTR remains the most important modifiable determinant of outcome across all subtypes; adjuvant radiotherapy should be reserved for subtotal resection, grade 3 histology, capsular violation in SP-MPE, or disseminated disease, while chemotherapy has no established frontline role outside salvage or infant-deferral settings [2, 3, 7, 9, 19, 28, 43, 44, 78]. Finally, lifelong surveillance imaging is warranted in all patients given the risk of both local and disseminated late recurrence [9, 19, 20]. 

Several key questions remain unresolved. Prospective multicenter studies are needed to clarify the optimal indications and timing for adjuvant radiotherapy after both total and subtotal resection instances, particularly in cases of myxopapillary ependymoma [54, 55, 71, 73, 74]. Additionally, the role of molecular stratification in tailoring individualized therapy—beyond identifying the rare MYCN-amplified high-risk subgroup—requires further validation [82]. The long-term functional and neurocognitive outcomes following combined modality therapy are not fully characterized, and there is an ongoing priority to develop novel systemic or targeted therapies for high-risk or recurrent disease [83]. The current literature is limited by retrospective designs, varying treatment protocols, and relatively small cohorts specific to spinal tumors, which limits confidence in definitive treatment algorithms.

## Supplementary Information

Below is the link to the electronic supplementary material.

Supplementary Material 1

## Data Availability

No datasets were generated or analysed during the current study.
