# Supplementary material for: Spinal ependymoma: a comprehensive review of molecular classification, management guidelines, and clinical outcomes with a focus on the pediatric population (Part III of ependymomas across compartments)
Source: J Neurooncol. 2026 Oct 6;180(1):6. doi: 10.1007/s11060-026-05816-9 (PMC13642508; doi:10.1007/s11060-026-05816-9)
Supplement: Supplementary file 1 [file 11060_2026_5816_MOESM1_ESM.docx]

**SUPPLEMENTARY TABLES AND FIGURES**

**Supplementary Table 1:** Landmark Papers in Pediatric Spinal Ependymoma

| Clinical Presentation and Preoperative Imaging | | | | | | | | | | | |
| --- | --- | --- | --- | --- | --- | --- | --- | --- | --- | --- | --- |
| **Author / Year** | **Study Type** | **Pediatric vs Mixed** | **Spinal vs CNS** | **Sample Size** | **Population / Scope** | **Extent of Resection Findings** | **RT Findings** | **Recurrence Patterns** | **Molecular / Pathology Notes** | **Management Implication** | **Key Limitation** |
| McGuire et al., 2009^90^ | SEER population analysis | Pediatric | All CNS sites | 635 | Children with ependymoma | Not a resection-focused study | RT use varied by location | Spinal tumors demonstrated improved survival | Predates molecular classification | Highlights prognostic importance of tumor location | Registry dataset |
| Amirian et al., 2012^91^ | SEER cohort study | Mixed | All CNS sites | 2688 | Pediatric and adult ependymoma | Not primary focus | RT associated with survival differences depending on location | Age and tumor location predicted survival | Limited molecular characterization | Supports risk stratification frameworks | Registry limitations |
| Marinoff et al., 2017^84^ | Multicenter retrospective cohort | Pediatric | Mostly intracranial | 463 | Pediatric ependymoma | Extent of resection associated with survival | RT commonly incorporated | High local recurrence rates reported | Limited molecular integration | Reinforces need for long-term surveillance | Mostly intracranial population |
| Tsang et al., 2020^71^ | International review | Pediatric | Spinal | NA | Pediatric spinal ependymoma | Synthesized multi-institutional management emphasizing maximal safe resection | Overview of RT indications after STR | Highlights recurrence patterns and need for surveillance | Limited granular molecular detail | Bridge between surgery and adjuvant therapy decisions | Review- format evidence |
| Operative Strategy: Achieving Safe Gross Total Resection | | | | | | | | | | | |
| **Author / Year** | **Study Type** | **Pediatric vs Mixed** | **Spinal vs CNS** | **Sample Size** | **Population / Scope** | **Extent of Resection Findings** | **RT Findings** | **Recurrence Patterns** | **Molecular / Pathology Notes** | **Management Implication** | **Key Limitation** |
| McCormick et al., 1990^92^ | Retrospective surgical series | Mixed (adult-dominant) | Spinal intramedullary | 23 | Intramedullary spinal ependymoma | Complete resection associated with durable disease control | Not primary focus | Long-term disease-free survival after complete resection | Predates contemporary molecular classification | Foundational support for GTR when safely feasible | Adult- dominant cohort |
| Bagley et al., 2007^54^ | Retrospective pediatric series | Pediatric | Spinal MPE | 14 | Children with myxopapillary ependymoma | Aggressive GTR favored when feasible | RT role uncertain | Recurrence risk persisted despite good outcomes | None | Supports en bloc GTR and lifelong surveillance | Small cohort |
| Benesch et al., 2010^66^ | Multicenter retrospective cohort | Pediatric | Spinal | 29 | Children/ adolescents with spinal ependymoma | Benefit of GTR demonstrated | Adjuvant therapy heterogeneous | 5‑year PFS favored GTR | Pre-methylation era | Pediatric anchor study for surgical outcomes | Historic treatment heterogeneity |
| Boström et al., 2011^4^ | Retrospective surgical series | Mixed | Spinal | NR | Spinal ependymoma | Extent of resection predicted outcomes | Not primary focus | Incomplete resection associated with worse tumor control | Limited molecular data | Supports maximal safe resection | Mixed-age cohort |
| Kucia et al., 2011^46^ | Surgical series | Mixed | Spinal | 67 | Intramedullary spinal tumors | GTR associated with improved outcomes | Not RT-focused | Recurrence lower after complete resection | None | Technique-focused surgical outcomes | Single center |
| Lee et al., 2013^49^ | Multicenter cohort | Mixed | Spinal | 88 | Spinal ependymoma | GTR strongest predictor of survival | RT selectively used | Durable tumor control after GTR | Minimal molecular data | Large spinal surgical dataset | Mostly adult population |
| Safaee et al., 2014^67^ | Retrospective pediatric/AYA series | Pediatric/AYA | Spinal | 24 | Children and young adults | Recurrence lower after GTR than STR | Not primary focus | Upper cord location carried higher recurrence risk | No molecular data | Supports aggressive but safe resection | Small cohort |
| Klekamp et al., 2015^48^ | Surgical review | Mixed | Spinal | NA | Intramedullary spinal tumors | Plane-based microsurgical resection emphasized | Not RT-focused | Discusses recurrence after STR | Limited molecular integration | Operative philosophy reference | Review article |
| Lundar et al., 2021^20^ | Long-term institutional series | Pediatric | Spinal | 33 | Children/adolescents | Extent of resection associated with outcomes | RT variable | Late recurrence documented | Limited molecular data | Supports lifelong surveillance | Era heterogeneity |
| Narin et al., 2024^2^ | Retrospective pediatric series | Pediatric | Spinal | NR | 25‑year pediatric institutional experience | Modern surgery-first outcomes | Adjuvant therapy selective | Contemporary recurrence data | Limited molecular profiling | Supports surgery-first management | Single institution |
| Pathology, Molecular Classification, and Risk Stratification | | | | | | | | | | | |
| **Author / Year** | **Study Type** | **Pediatric vs Mixed** | **Spinal vs CNS** | **Sample Size** | **Population / Scope** | **Extent of Resection Findings** | **RT Findings** | **Recurrence Patterns** | **Molecular / Pathology Notes** | **Management Implication** | **Key Limitation** |
| Carter et al., 2002^93^ | Genetic abnormality study | Mixed | CNS | 86 | Ependymoma tumors | Not a resection-outcomes study | Not RT-focused | Not primary focus | Chromosomal abnormalities including 22q loss | Early molecular insights | Pre-methylation era |
| Barton et al., 2010^94^ | Molecular characterization study | Mixed | Pediatric MPE emphasis | 29 | Pediatric myxopapillary ependymoma | Not surgical | Not treatment-defining | Exploratory biologic findings | Distinct gene expression patterns | Supports concept MPE biologically distinct | Exploratory study |
| Kilday et al., 2009^95^ | Biologic review | Pediatric | CNS | NA | Pediatric ependymoma | Not surgical focus | RT discussed broadly | Discusses recurrence risk | Genomic differences pediatric vs adult | Conceptual disease biology framework | Review |
| Pajtler et al., 2015^26^ | DNA methylation classification | Mixed | All CNS compartments | 500 | Ependymal tumors | Not surgical study | Not treatment-focused | Refined prognostic subgrouping | Defined modern molecular classes | Foundation of modern classification | Not management-directed |
| Witt et al., 2018^96^ | Methylation study | Adult-focused | All CNS | 122 | Adult ependymoma | Not surgical | Not RT-focused | Improved diagnostic interpretation | Validated methylation groups | Supports molecular diagnostics | Adult cohort |
| Ghasemi et al., 2019^15^ | Molecular pathology study | Mixed | Spinal | NR | MYCN amplified spinal ependymoma | Not surgical | Not RT-focused | Early aggressive relapse | Defined MYCN amplified subtype | Identifies high-risk disease | Rare subtype |
| Raffeld et al., 2020^68^ | Molecular confirmation study | Mixed | Spinal | NR | MYCN amplified tumors | Not surgical | Not RT-focused | Aggressive clinical behavior | Confirmed distinct methylation signature | Supports reflex MYCN testing | Rare cohort |
| Engertsberger et al., 2024^13^ | Pediatric molecular prognostic cohort | Pediatric/AYA | Spinal | 83 | HIT‑MED spinal cohort | Extent of resection and grade dominate prognosis | RT variable | MYCN subgroup early relapse | Best pediatric spinal molecular dataset | Supports surgery-first paradigm | Small MYCN subgroup |
| Pohl et al., 2024^17^ | Large molecular survival model | Mixed | All CNS | 2023 | All-compartment ependymoma | Not surgical | Not RT-specific | Improved survival prediction | Integrates modern molecular classification | Supports contemporary prognostic framing | Not spinal-specific |
| Postoperative Management, Recurrence Patterns, and Surveillance | | | | | | | | | | | |
| **Author / Year** | **Study Type** | **Pediatric vs Mixed** | **Spinal vs CNS** | **Sample Size** | **Population / Scope** | **Extent of Resection Findings** | **RT Findings** | **Recurrence Patterns** | **Molecular / Pathology Notes** | **Management Implication** | **Key Limitation** |
| Merchant et al., 2009^72^ | Prospective RT trial | Pediatric | Mostly intracranial | 153 | Localized pediatric ependymoma | Extent of resection predicted outcomes | Defined conformal RT principles | Improved local control and survival | Not spinal-specific | Supports RT timing principles | Mostly intracranial |
| Merchant et al., 2019^78^ | Prospective protocol study | Pediatric/AYA | Mostly intracranial | 356 | Pediatric ependymoma | Reinforced importance of local control | Clarified RT and chemotherapy roles | Chemotherapy limited first-line role | Limited spinal molecular application | Supports limited chemotherapy role | Intracranial dominant |
| Rudà (EANO) et al., 2018^9^ | Guideline | Mixed | All CNS | NA | Ependymal tumors | Consensus emphasis on maximal safe resection | Defines RT indications | Discusses recurrence and surveillance | Incorporates modern classification | Standard consensus framework | Not spinal-specific |
| Simón et al., 2025^19^ | European practice recommendations | Pediatric/AYA | All CNS | NA | Newly diagnosed pediatric ependymoma | Surgery-first risk-adapted framework | Modern RT decision framework | Surveillance recommendations | Integrates molecular classification | Most current pediatric practice guidance | Not spinal-only |
| Abbreviations: AYA = adolescent/young adult; CNS = central nervous system; GTR = gross total resection; MPE = myxopapillary ependymoma; NR = not reported; RT = radiotherapy. | | | | | | | | | | | |

**Supplementary Case Presentations**

*Case 1: Spinal Ependymoma (SP-EPN)*

A 49-year-old male presented with progressive lower extremity weakness, numbness, pain, paresthesias, and bowel/bladder incontinence after an extensive, unrevealing workup. MRI of the neuraxis demonstrated an enhancing intramedullary spinal cord lesion centered at T5 with mild cord expansion, surrounding edema, marginal hemosiderin/blood products, and a small associated distal thoracic syrinx **(Figure 2)**. The remainder of the neuraxis imaging was negative. Given the irregular appearance of the lesion, spinal angiography was obtained preoperatively and was negative for an intramedullary vascular lesion. Given the patient’s progressive neurologic decline and bowel/bladder dysfunction, he underwent T4–T6 laminoplasty for microsurgical resection of the intramedullary lesion. Intraoperatively, the spinal cord was expanded at the T5 level with yellow discoloration consistent with the seen hemosiderin staining. A myelotomy was performed over the thinnest portion of the cord, and abnormal intramedullary tissue was exposed. The lesion was densely adherent to the surrounding spinal cord parenchyma, requiring careful circumferential dissection. Frozen pathology was consistent with ependymoma. Postoperative MRI demonstrated no residual enhancing lesion, consistent with gross total resection **(Figure 2)**. Follow-up still ongoing.

*Case 2: Spinal Myxopapillary Ependymomas (SP-MPE)*

A previously healthy 17-year-old male presented with 5 months of progressive lower mechanical back pain and proximal leg weakness. An MRI demonstrated a large T2 hyperintense, enhancing intradural extramedullary solid cystic mass extending from T11-S2 **(Figure 3)**. A T11-L5 laminoplasty, S1 and S2 laminectomy for microsurgical resection of an intradural extramedullary tumor was completed **(Figure 3).** The tumor was very adherent to the lumbosacral nerve roots **(Figure 4)**. The laminoplasty was secured with sutures. Pathology was consistent with a Myxopapillary Ependymoma SP-MPE (WHO Grade 2). It was characterized by moderately papillary structures arranged around myxoid cores with moderate hyalinization and frequent perivascular pseudorosettes. It was positive for GFAP, negative for EMA, and positive for S-100. Alcian Blue stain highlighted abundant myxoid material. Postoperatively, the MRI demonstrated a gross total resection, and the patient's preoperative back pain was notably improved. He received focal radiation for a total of 50.4 Gy. MRI at 3 and 6 months postoperatively demonstrated no evidence of recurrence.
